# Effect of Veneering Porcelain Thickness and Background Substrate on the Color Matching of Zirconia-Based Restorations: An In-Vitro Study

**DOI:** 10.21142/2523-2754-1404-2026-311

**Published:** 2026-10-01

**Authors:** Yasaman Yazdandoust, Delaram Ghaemi, Mohamad Reza Nakhaee, Arsalan Shahri, Saeed Akhlaghi, Sobhan Ghaemi

**Affiliations:** 1 School of Public Health, University of Alberta. Edmonton, Canada. yasamanyazdandoust@gmail.com University of Alberta School of Public Health University of Alberta Edmonton Canada yasamanyazdandoust@gmail.com; 2 Department of Prosthodontics, Mashhad University of Medical Sciences. Mashhad, Iran. ghaemid@mums.ac.ir Mashhad University of Medical Sciences Department of Prosthodontics Mashhad University of Medical Sciences Mashhad Iran ghaemid@mums.ac.ir; 3 Department of Prosthodontics, School of Dentistry, Mashhad University of Medical Sciences. Mashhad, Iran. NakhaeeMR@mums.ac.ir Mashhad University of Medical Sciences Department of Prosthodontics School of Dentistry Mashhad University of Medical Sciences Mashhad Iran NakhaeeMR@mums.ac.ir; 4 Dental Materials Research Center, Mashhad Dental School, Mashhad University of Medical Sciences. Mashhad, Iran. Arsalshahri98@gmail.com Mashhad University of Medical Sciences Dental Materials Research Center Mashhad Dental School Mashhad University of Medical Sciences Mashhad Iran; 5 Department of Biostatistics, School of Health, Mashhad University of Medical Sciences. Mashhad, Iran. akhlaghis@mums.ac.ir Mashhad University of Medical Sciences Department of Biostatistics School of Health Mashhad University of Medical Sciences Mashhad Iran akhlaghis@mums.ac.ir; 6 Periodontics Department, Faculty of Dentistry, Islamic Azad University. Tehran, Iran. Sobhan.ghaemi73@gmail.com Islamic Azad University Periodontics Department Faculty of Dentistry Islamic Azad University Tehran Iran Sobhan.ghaemi73@gmail.com

**Keywords:** dental porcelain, esthetics, CIELAB, composite resins, porcelana dental, estética, CIELAB, resinas compuestas

## Abstract

**Objective:**

Zirconia-based restorations are increasingly used in esthetic dentistry due to their mechanical strength and favorable optical properties. However, challenges in achieving accurate color matching remain, particularly due to the influence of veneering porcelain thickness and background shade. This study aimed to evaluate the effect of these two variables on the final color of zirconia restorations.

**Materials and methods:**

This in vitro study evaluated 33 zirconia discs (A2 shade, 0.5 mm thick), each veneered with porcelain to final thicknesses of 0.8 mm, 1.0 mm, or 1.2 mm (n = 11 per group). Each specimen was measured over three background materials: composite resin, titanium, and nickel-chromium. CIELAB coordinates were recorded using a colorimeter, and color differences (∆E₀₀) relative to the VITA reference shade were calculated using the CIEDE2000 formula. Data were analyzed using two-way repeated-measures ANOVA followed by Tukey’s post hoc test.

**Results:**

Both porcelain thickness and background shade significantly affected L*, a*, and ∆E₀₀ values (p < 0.01). Thickness had no significant effect on b* values, while background shade did. The 1.0 mm porcelain group achieved the most favorable ∆E₀₀ values across all backgrounds, with titanium consistently yielding the poorest color match.

**Conclusion:**

Porcelain thickness and background shade significantly influence the final color of zirconia restorations. A veneering thickness of 1.0 mm provided the most acceptable shade matching, particularly over composite and Ni-Cr substrates. These findings underscore the importance of optimizing both material layering and background management in clinical practice.

## Resumen

**Objetivo:**

Las restauraciones a base de zirconia se utilizan cada vez más en la odontología estética debido a su resistencia mecánica y sus favorables propiedades ópticas. Sin embargo, persisten desafíos para lograr una adecuada coincidencia de color, particularmente debido a la influencia del espesor de la porcelana de recubrimiento y el tono del sustrato. El objetivo de este estudio fue evaluar el efecto de estas dos variables sobre el color final de las restauraciones de zirconia.

**Materiales y métodos:**

Este estudio in vitro evaluó 33 discos de zirconia (tono A2, 0,5 mm de espesor), recubiertos con porcelana hasta alcanzar espesores finales de 0,8 mm, 1,0 mm o 1,2 mm (n = 11 por grupo). Cada espécimen fue medido sobre tres materiales de fondo: resina compuesta, titanio y níquel-cromo. Las coordenadas CIELAB se registraron mediante un colorímetro y las diferencias de color (∆E₀₀) respecto del tono de referencia VITA se calcularon utilizando la fórmula CIEDE2000. Los datos se analizaron mediante ANOVA de medidas repetidas de dos factores, seguido de la prueba post hoc de Tukey.

**Resultados:**

Tanto el espesor de la porcelana como el tono del sustrato influyeron significativamente en los valores L*, a* y ∆E₀₀ (p < 0,01). El espesor no mostró un efecto significativo sobre los valores b*, mientras que el tono del sustrato sí lo hizo. El grupo con porcelana de 1,0 mm presentó los valores más favorables de ∆E₀₀ en todos los sustratos, siendo el titanio el que mostró consistentemente la peor coincidencia de color.

**Conclusión:**

El espesor de la porcelana y el tono del sustrato influyen significativamente en el color final de las restauraciones de zirconia. Un espesor de recubrimiento de 1,0 mm proporcionó la coincidencia de color más aceptable, especialmente sobre sustratos de resina compuesta y Ni-Cr. Estos hallazgos resaltan la importancia de optimizar tanto la estratificación del material como el manejo del sustrato en la práctica clínica.

## INTRODUCTION

Porcelain-fused-to-metal (PFM) restorations have long been a standard in dental prosthetics, valued for their mechanical strength and long-term durability. However, limitations in color matching, particularly in the esthetic zone, remain a concern due to the opacity and optical interference of the underlying metal substructure. ^1^ To address these shortcomings, all-ceramic restorations have become increasingly favored by clinicians, largely due to their translucent cores and superior esthetic potential. ^2^

Among all-ceramic materials, zirconia stands out for its high strength, favorable optical properties, and acceptable durability. ^3^ Classified as a semi-translucent ceramic, zirconia demonstrates a wide range of translucency values, which vary significantly based on its composition (yttria content) and thickness ^4^. It is also highly biocompatible and possesses the highest fracture toughness and flexural strength among all ceramics. ^5^ Furthermore, Zirconia’s high fracture toughness is primarily attributed to its stress-induced transformation toughening mechanism, in which metastable tetragonal zirconia grains transform into the monoclinic phase at crack tips, producing a localized volume expansion that compresses and slows crack propagation. ^6^

Clinically, zirconia restorations are categorized into two main types: monolithic and veneered. In veneered systems, a high-strength zirconia coping is layered with porcelain to enhance esthetics, combining structural durability with a lifelike appearance. Despite their optical advantages over PFMs, zirconia-based restorations still present challenges in achieving consistent shade matching. Multiple factors, including the shade and thickness of the zirconia core, veneering ceramic, background substrate, and luting cement, interact to influence the final color outcome. ^7^ Recently, the interplay between background shade and restoration thickness is emphasized in ultra-translucent zirconia systems, particularly for anterior restorations where subtle shade discrepancies are more visible. ^8^

The color outcome of zirconia-based restorations is influenced by the characteristics of all restorative layers, including the background, cement, coping, and veneering ceramic. ^7^ Factors such as zirconia core type, brand, color, and thickness, as well as the translucency and application technique of the veneering ceramic, collectively determine the final shade. ^7^ One study demonstrated that increasing ceramic layer thickness enhances masking ability by reducing translucency, emphasizing the critical role of veneer thickness in achieving accurate shade matching. ^8^ Another investigation reported that a veneering thickness of at least 1.5 mm is necessary to obtain acceptable color matching across diverse abutment materials, highlighting the clinical relevance of optimized material layering. ^9^ More recent evidence showed that even ultra-translucent zirconia systems are significantly influenced by the combined effects of veneer thickness and cement shade, underscoring the need for precise control of all restorative layers to reproduce the optical behavior of natural teeth. ^10^

Zirconia restorations are increasingly used due to their mechanical reliability and esthetic performance; however, variability in the translucency of veneered zirconia systems limits predictable reproduction of natural tooth color. ^11^ Though evidence indicates that increasing veneering ceramic thickness enhances masking of dark substrates ^12^, it may exceed clinically acceptable thickness ranges. Accordingly, this in vitro study evaluated the influence of veneering porcelain thickness and background shade on the color match of zirconia-based restorations to determine clinically relevant thresholds for esthetic acceptability. The null hypothesis was that neither veneering porcelain thickness nor background shade would significantly affect the final color of all-ceramic restorations.

## METHODS AND MATERIALS

This experimental in vitro study was approved by the Ethics Committee of Mashhad University of Medical Sciences (IR.MUMS.DENTISTRY.REC.1399.076).

### Sample size calculation

Based on Tabatabaian et al. ^13^, using an alpha of 0.05, 95% confidence intervals, and 80% power, the minimum sample size in this equivalence study was 11 samples in each group to be able to reject the null hypothesis. The Type I error probability associated with this test of this null hypothesis is 0.05. Sample size calculation was performed using G*Power, version 3.1.9.6 for MS Windows (Franz Faul, Universität Kiel, Germany).

A total of 33 zirconia discs (12×12 mm², 0.5 mm thickness) were fabricated and allocated randomly into three groups of 11 specimens each. All discs were milled from pre-colored A2 zirconia blocks (inCoris TZI C; Dentsply Sirona, Germany) using a computer-aided design/computer-aided manufacturing (CAD/CAM) system (CORiTEC 250i, imes-icore GmbH, Eiterfeld, Germany). Given that zirconia translucency is thickness-dependent, precise control over specimen thickness was essential. To account for post-sintering shrinkage, the discs were initially milled at 0.6 mm thickness to achieve a final thickness of 0.5 mm after sintering, as per the manufacturer’s recommendations.

Sintering was performed using a high-temperature furnace (Speed HTC inFire, Dentsply Sirona, Germany). Following sintering, disc thicknesses were verified with a digital caliper (INSIZE-1108.150A, China; accuracy ± 0.01 mm), confirming a range of 0.54 ± 0.04 mm. Prior to veneering, specimens were immersed in 96% ethanol for 15 minutes and dried using an air syringe.

The discs were veneered with 2M2 Transpa Dentine porcelain (VITA VM9, VITA Zahnfabrik, Germany) using a manual layering technique. According to the VITA VM13 porcelain application protocol, excess porcelain was applied to compensate for shrinkage, targeting final veneer thicknesses of 0.8 mm, 1.0 mm, and 1.2 mm across the three groups. Firing was conducted over 10 minutes, heating from 500 °C to 910 °C at a rate of 55 °C/min. After natural cooling to room temperature, all specimens were polished and adjusted to a precision of ± 0.02 mm using a porcelain adjustment kit (SHOFU, Japan). Final veneer thicknesses were re-confirmed using the digital caliper. All procedures were performed by a single trained operator.

Each disc was evaluated against three background materials:

- Titanium alloy (Ti): Fabricated by milling Grade-4 titanium discs (imes-icore GmbH, Germany). All background specimens measured 10 mm in diameter and 15 mm in thickness.
- A2-shade light-cured composite resin: A2 composite resin (3M ESPE, USA), molded into 15 mm-thick cylindrical forms, and polymerized incrementally for 40 seconds using an 800 mW/cm² light-curing unit (Bluephase C8, Ivoclar Vivadent, Liechtenstein).
- Nickel-chromium alloy (Ni-Cr): Fabricated by casting the alloy into cylindrical wax patterns and polished using a fine green rubber bur (Airgoeresin, USA).

For optical measurement, each veneered disc was immersed again in 96% ethanol for 15 minutes prior to colorimetry. Color values were recorded using a colorimeter (Chroma Meter CR-400, Konica Minolta, Japan) under standardized D65 lighting conditions and a 2° observer angle. The device was calibrated according to the manufacturer’s instructions before each session. CIELAB values of the background materials were also recorded as follows:

- Ti: L* = 62.57, a* = 0.43, b* = 7.06
- Composite: L* = 74.54, a* = -0.18, b* = 17.65
- Ni-Cr: L* = 70.24, a* = 0.25, b* = 5.44

To eliminate external light and standardize positioning, putty material (Speedex Putty, Coltene, Switzerland) was shaped into molds at the colorimeter’s opening to hold each specimen firmly against its respective background. CIELAB values were recorded at the center of each specimen three times per background, and the mean value was used for analysis. The optical values of the 2M2 shade from the VITA Toothguide 3D-MASTER® were also measured at the center of its middle third (L* = 70.21, a* = 1.5, b* = 13.91) and served as the reference shade.

Color differences (∆E₀₀) between the reference shade and each specimen across backgrounds and thicknesses were evaluated using the CIEDE2000 metric, with threshold values of 0.8 for perceptibility and 1.8 for acceptability ^14^:

∆E_{_₀₀_}_ = 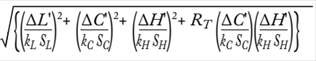


All weighting parameters were set to 1 (kL = kC = kH = 1), as recommended by Durand et al. ^15^. ∆E₀₀ values were computed using the Colormine.org online tool.

All the statistical analyses were performed with the significance level set at 5%. Data were analyzed using SPSS version 21 (SPSS Inc., Chicago, IL, USA). The normality of data distribution was assessed using the one-sample Kolmogorov-Smirnov test. Because each specimen was measured on all three backgrounds, a two-way repeated-measures ANOVA was used, with veneering thickness as a between-specimen factor and background as a within-specimen factor. When a significant main effect was detected, pairwise comparisons were performed with Tukey’s post-hoc test. Assumptions were checked, and the interaction term between thickness and background was also evaluated.

## RESULTS

All data were normally distributed (Kolmogorov-Smirnov, p > 0.05). Mean (±SD) values for L*, a*, b*, and ∆E₀₀ across porcelain thicknesses (0.8, 1.0, 1.2 mm) and backgrounds (composite, titanium, Ni-Cr) are presented in Table 1. The two-way repeated-measures ANOVA showed no significant interaction between veneering thickness and background for any color parameter (p > 0.05); therefore, only the main effects of thickness and background are reported.

**Table 1:** Mean ± SD of CIELAB coordinates (L*, a*, b*) and color difference (ΔE00) of veneered zirconia restorations by porcelain thickness and background, relative to the VITA reference shade

Variable	Background	Mean ± SD			Two-way ANOVA		
		0.8 mm	1.0 mm	1.2 mm	Thickness	Background	Interaction
L*	Composite A	68.17 ± 1.44 a	70.20 ± 0.57 b	70.26 ± 0.87 b	p < 0.01	p < 0.01	p = 0.91
	Titanium B	67.38 ± 1.15 a	69.29 ± 0.63 b	69.49 ± 1.44 b	p < 0.01	p < 0.01	p = 0.91
	Nickel-Chromium B	67.22 ± 1.76 a	69.55 ± 0.61 b	69.87 ± 0.66 b	p < 0.01	p < 0.01	p = 0.91
a*	Composite A	1.76 ± 0.20 a	2.14 ± 0.10 b	2.10 ± 0.11 b	p < 0.01	p < 0.01	p = 0.33
	Titanium B	1.21 ± 0.13 a	1.57 ± 0.11 b	1.61 ± 0.20 b	p < 0.01	p < 0.01	p = 0.33
	Nickel-Chromium C	1.25 ± 0.13 a	1.71 ± 0.10 b	1.76 ± 0.13 b	p < 0.01	p < 0.01	p = 0.33
b*	Composite A	14.39 ± 2.30 a	14.19 ± 0.31 a	13.87 ± 1.35 a	p = 0.34	p < 0.01	p = 0.99
	Titanium B	13.00 ± 1.81 a	13.04 ± 0.32 a	12.99 ± 1.35 a	p = 0.34	p < 0.01	p = 0.99
	Nickel-Chromium B	13.41 ± 2.01 a	13.36 ± 0.33 a	13.28 ± 1.42 a	p = 0.34	p < 0.01	p = 0.99
∆E_00_	Composite A	2.05 ± 1.23 a	0.87 ± 0.17 b	1.16 ± 0.60 c	p < 0.01	p = 0.01	p = 0.10
	Titanium B	3.69 ± 2.42 a	0.94 ± 0.44 b	1.96 ± 1.72 c	p < 0.01	p = 0.01	p = 0.10
	Nickel-Chromium AB	2.80 ± 1.23 a	0.81 ± 0.30 b	1.05 ± 0.46 c	p < 0.01	p = 0.01	p = 0.10

Different uppercase letters indicate a statistically significant difference between backgrounds for each variable according to the Tukey’s post-hoc test.

Different lowercase letters indicate a statistically significant difference between thickness levels within the same background according to the Tukey’s post-hoc test.

### L*

Two-way ANOVA (mixed-model) revealed significant effects of both thickness and background (p < 0.01), with no interaction (p = 0.91). Tukey’s test showed significant differences between 0.8 mm and both 1.0 mm and 1.2 mm (p < 0.01); no difference between 1.0 mm and 1.2 mm (p = 0.73). Among backgrounds, composite differed significantly from titanium (p = 0.02) and Ni-Cr (p = 0.04); titanium vs. Ni-Cr was not significant (p = 0.93). L* increased with thickness across all backgrounds.

### a*

Significant effects were observed for thickness and background (p < 0.01); the interaction was not significant (p = 0.33). Differences were found between 0.8 mm and both 1.0 mm and 1.2 mm (p < 0.01); 1.0 mm vs. 1.2 mm was not significant (p = 0.87). Composite differed from titanium (p < 0.01) and Ni-Cr (p = 0.01); also, titanium vs. Ni-Cr was significant (p = 0.01). a* increased with thickness.

### b*

Only background had a significant effect (p < 0.01); thickness (p = 0.34) and interaction (p = 0.99) were not significant. No significant differences were found among thickness groups (all p = 0.35). Composite differed from titanium (p < 0.01) and Ni-Cr (p = 0.03); titanium vs. Ni-Cr was not significant (p = 0.45). b* decreased with thickness on composite and Ni-Cr; remained stable on titanium.

### ∆E₀₀

Thresholds for perceptibility and acceptability were set at 0.8 and 1.8. Both thickness and background significantly affected ∆E₀₀ (p < 0.01 and p = 0.01, respectively); no interaction was found (p = 0.10). Tukey’s test showed significant differences between all thickness pairs (p < 0.01). Only composite vs. titanium was significant among backgrounds (p = 0.02); other comparisons were not (p ≥ 0.05). In all three backgrounds, the ∆E₀₀ value decreased from 0.8 mm to 1.0 mm and increased from 1.0 mm to 1.2 mm (Figure 1).

**Figure 1:**
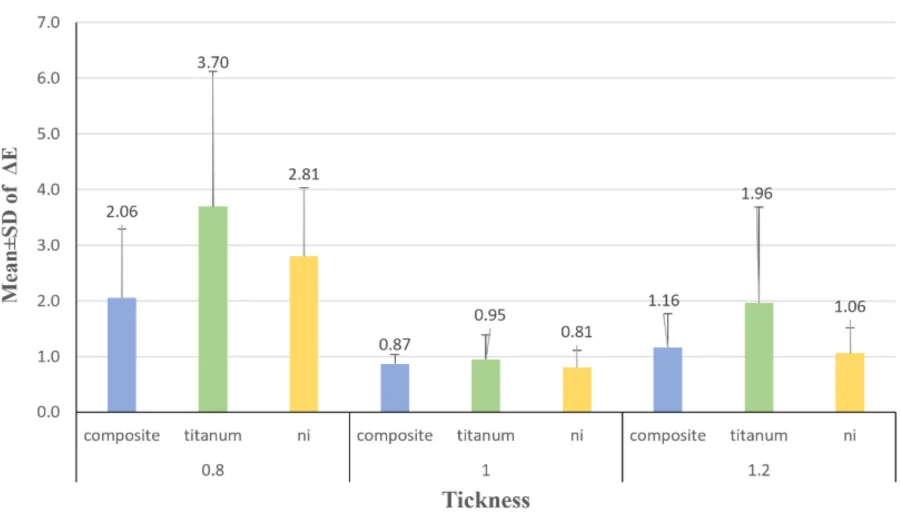
Mean ± SD of ∆E_00_ by veneering thickness (0.8, 1.0, 1.2 mm) and background.

## DISCUSSION 

In this study, both background type and porcelain thickness significantly influenced the CIELAB components and ∆E₀₀ values, with thickness affecting all variables except for b*. Statistically significant differences were observed between 0.8 mm and both 1.0 mm and 1.2 mm thicknesses across all variables, excluding b*. Among background comparisons, composite and titanium demonstrated significant differences in all tested parameters. The CIEDE2000 formula, introduced by the CIE in 2001, is the most widely used method for quantifying color differences, offering improved alignment with human visual perception compared to the earlier CIELAB formula. ^16^ Based on its established perceptibility (∆E₀₀ = 0.8) and acceptability (∆E₀₀ = 1.8) thresholds ^14^, a porcelain thickness of 1.0 mm across all backgrounds and 1.2 mm on composite and nickel-chromium backgrounds yielded clinically acceptable color matching to the target shade (2M2). 

As a semi-translucent ceramic, zirconia allows partial light transmission through its structure. ^17^ The underlying background can show through the zirconia substructure and alter the perceived color at the surface. ^13^ Consequently, the final shade results from the combined contributions of the ceramic and the background (and, clinically, the cement), rather than the ceramic alone. ^7^ Considering the results of this study, it can be concluded that in metallic substrates like titanium, light is absorbed and reflected at the surface, rendering them highly opaque with strong reflective properties. This results in the metallic hue being projected through the semi-translucent ceramic layers. In contrast, the nickel-chromium alloy, fabricated via casting, is more porous and less polished than titanium, resulting in lower reflectivity. This may account for the distinct optical behavior of Ni-Cr, which, despite being metallic, did not produce a statistically significant ∆E₀₀ difference from the composite background.

Based on the results of this study, in non-metallic materials such as composites, light scattering and transmission occur within both the interface and the bulk of the material. As a result of refraction, scattering predominates over reflection, thereby diminishing the substrate’s optical influence on the final color of ceramic restorations. This varying degree of optical interaction becomes more critical when there is a substantial color difference between the substrate and the intended shade. ^7^^,^^18^ In this study, the titanium background exhibited the highest color difference from the target shade (∆E₀₀ = 7.78), resulting in the poorest color match across all porcelain thicknesses. Increasing porcelain thickness was shown to reduce the influence of the background. Specifically, increasing thickness from 0.8 mm to 1.0 mm improved color matching, consistent with another previous study. ^19^ However, further increasing the thickness to 1.2 mm led to a rise in ∆E, potentially due to greater light scattering and refraction across thicker layers. As more light is scattered and absorbed through increasing porcelain thickness, less is reflected from the opaque core, contributing to elevated ∆E values as corroborated by previous studies. ^20^^,^^21^

Although ∆E₀₀ values across all backgrounds remained above the perceptibility threshold, the most favorable results were observed at a 1.0 mm thickness, with values of 0.81, 0.87, and 0.95 for Ni-Cr, composite, and titanium backgrounds, respectively. These values are marginally above the threshold, suggesting potential clinical acceptability. Furthermore, titanium abutments are more frequently used in implant-supported restorations, where achieving sufficient porcelain thickness is typically more feasible than with natural dentition. ^22^

An increase in thickness corresponded with higher L* values across all backgrounds, resulting in a lighter appearance. a* values also increased, indicating a shift toward red, consistent with findings by Son et al. ^21^ In composite and Ni-Cr backgrounds, b* values decreased with increased thickness, indicating a shift toward blue, whereas b* remained relatively stable on titanium. The lower b* values observed in darker, metallic backgrounds highlight a more pronounced blue hue, as also reported by Al Hamad et al. ^23^ This suggests that red and blue hues become more prominent at greater thicknesses, which may be attributed to wavelength-dependent scattering. Shorter wavelengths in the visible spectrum (e.g., blue) scatter more intensely, and changes in reflectivity associated with thickness are more pronounced at shorter wavelengths. The human visual system is particularly sensitive to these wavelengths, further amplifying this effect.

The Vita 3D Master shade guide was selected due to its closer alignment with the 2M2 porcelain powder. ^21^ Spectrophotometers measure reflected light energy across 1 to 25 nm intervals in the visible spectrum and are known for their higher accuracy and objectivity compared to visual methods or conventional tools. ^24^ In contrast, colorimeters separate visible light into red, green, and blue channels and report tristimulus values (X, Y, Z), which are then graphically represented on a standard CIE color chart. Since colorimeters do not capture full spectral reflectance, their accuracy is generally lower than that of spectrophotometers. ^19^^,^^20^^,^^25^

Differences in L* were identified as the primary source of color mismatch between porcelain specimens and shade tabs. In the study by Son et al., increasing porcelain thickness resulted in higher a* and b* values but lower L*, producing clinically unacceptable ∆E₀₀ values in most 0.5 mm porcelain veneers. ^21^ Our study similarly demonstrated that porcelain thicknesses below 1 mm did not yield acceptable ∆E₀₀ values. However, in contrast to Son et al. ^21^, our study showed an increase in L* values, which may be attributed to differences in ceramic systems, measurement instrumentation (e.g., spectroradiometer), or surface treatments such as polishing.

Several studies have examined how porcelain thickness and background type influence color outcomes, yet their findings vary depending on materials, measurement devices, and research objectives. Sinmazisik et al. ^26^ reported that increasing porcelain thickness reduced color coordinates and produced perceptible ∆E₀₀ values at 1.5 mm. In contrast, our study found increased color coordinates and near-threshold ∆E₀₀ at 1.0 mm. These differences may be attributed to variations in ceramic systems, background darkness, and measurement tools (spectrophotometer vs. colorimeter). Notably, both studies highlight the sensitivity of color outcomes to thickness; however, our results suggest that clinically relevant thicknesses may behave differently than thicker layers evaluated in previous work.

When considering the influence of background shade, our findings diverge from those of Suputtamongkol et al. ^12^, who observed no significant effect of metallic or composite backgrounds on L*, a*, or b* coordinates, despite changes in overall perceived color. In our study, background type significantly influenced all measured variables. This discrepancy may stem from methodological differences, such as their use of natural teeth in intraoral conditions and cast post materials, as well as their specific focus on color masking rather than direct color coordinate shifts. Together, these contrasting results suggest that the interaction between background color and ceramic is highly dependent on the experimental setup and substrate characteristics.

The importance of adequate masking is further highlighted by Tabatabaian et al. ^25^, who reported minimum porcelain thickness requirements ranging from 1.6 mm to 2.0 mm depending on the background. Although informative, these values exceed the maximum clinically feasible porcelain thickness of approximately 1.5 mm, limiting their practical application. Our findings, which demonstrate noticeable background influence even at 1.0 mm, reinforce the challenge of achieving effective masking within realistic clinical constraints.

Collectively, these results align with the clinical insights summarized by Pekkan et al. ^20^, who emphasized that increased translucency enhances background influence, particularly in thin restorations (0.5-0.9 mm). The convergence of these studies supports the conclusion that as ceramic thickness decreases and material translucency increases; background color exerts a progressively stronger effect on both color coordinates and ∆E₀₀ outcomes. This underscores the need to carefully balance ceramic thickness and substrate shade when planning esthetic restorations.

Saravi et al. ^27^ conducted a meta-analysis of CAD/CAM-fabricated all-ceramic fixed dental prostheses, identifying veneering layer chipping as a major technical complication, particularly in layered zirconia systems. Their 10-year survival analysis revealed a decline in esthetic performance and integrity over time, underscoring the need to optimize veneering thickness and substrate compatibility to reduce ∆E₀₀ variability. These conclusions are consistent with our findings, which suggest that a 1.0 mm porcelain thickness offers a favorable compromise between esthetic masking and material performance, especially over Ni-Cr and composite substrates.

Similarly, Woo et al. ^9^ demonstrated that a minimum veneering thickness of 1.5 mm is necessary to achieve ∆E₀₀ values below the clinical acceptability threshold when zirconia restorations are placed over metal or zirconia abutments. Their findings emphasized the critical role of substrate color, with titanium backgrounds consistently yielding higher ∆E₀₀ values, a trend also confirmed in our study. They further noted that highly translucent materials such as Prettau Anterior zirconia, although visually appealing, may inadequately mask dark substrates at minimal thicknesses, reinforcing the need for material-specific thickness guidelines.

Consistent with our results, Mekled et al. ^28^ found that 0.75 mm and 1.0 mm veneering thicknesses provided more predictable shade outcomes. This supports our conclusion that a 1.0 mm porcelain thickness represents a clinically relevant threshold for achieving acceptable esthetics. While current study utilized a calibrated colorimeter, the spectrophotometric methodology employed by Mekled et al. ^28^ allows for finer spectral resolution.

All of the aforementioned studies utilized the CIE76 formula, which is considered less precise than the CIEDE2000 model employed in our study ^29^. While CIE76 offers a basic quantitative approach, CIEDE2000 improves perceptual agreement by correcting CIELAB’s non-uniformities via weighting functions (SL, SC, SH), parametric factors (KL, KC, KH) for observational/experimental conditions, and a rotation term (RT) that models hue-chroma interactions, especially in the blue region ^16^. CIEDE2000 is now the standard in dental research for detecting subtle color differences with greater precision. ^30^

The study by Xu et al. ^31^ further demonstrated that increasing porcelain thickness improves color matching in metal-ceramic systems. Natural tooth color is predominantly determined by dentin reflection, which is modulated by enamel thickness, translucency, and scattering properties. Enhanced optical behavior in all-ceramic systems improves the contribution of dentin to the final shade. When the dentin layer exceeds the thickness of enamel, the final shade of zirconia restorations more closely resembles the desired target. The authors also selected the Vitapan Classical shade tab due to its widespread acceptance in clinical dental practice.

One limitation of this study is its in vitro design, which may not fully capture the complexities of clinical conditions. The observed ∆E₀₀ values across varying porcelain thicknesses and background materials do not account for intraoral factors such as restoration site, cement type and shade, or the glazing process, all of which can significantly influence final color perception. Additionally, the use of square-shaped specimens with uniform thickness fails to replicate the anatomical variability of actual restorations. Fabricating specimens as anatomically shaped copings would have allowed for a more clinically relevant assessment, as real-world restorations exhibit varying thicknesses that affect perceived color. Despite these limitations, the study's strength lies in its rigorous control of color-affecting variables, allowing for consistent and reliable comparison of final color outcomes under standardized conditions.

## CONCLUSION

Within the limitations of this in vitro study, both the background substrate and the veneering porcelain thickness shaped the color match (∆E₀₀) of zirconia restorations. A 1.0-mm veneer produced the lowest ∆E₀₀ across backgrounds; 0.8-mm did not reach acceptability. Titanium backgrounds performed worst at all thicknesses, compared with composite and Ni-Cr. Greater thickness raised L* and a*, making the result lighter and a bit redder; b* was determined chiefly by the background. In practice, aim for 1.0-mm veneers and plan additional masking when titanium is involved.
